# Femoral antecurvation—A 3D CT Analysis of 1232 adult femurs

**DOI:** 10.1371/journal.pone.0204961

**Published:** 2018-10-09

**Authors:** Darius M. Thiesen, Felix Prange, Josephine Berger-Groch, Dimitris Ntalos, Andreas Petersik, Bernhard Hofstätter, Johannes M. Rueger, Till O. Klatte, Maximilian J. Hartel

**Affiliations:** 1 Department of Trauma, Hand, and Reconstructive Surgery, University Medical Centre Hamburg-Eppendorf, Hamburg, Germany; 2 Stryker Trauma GmbH, Kiel-Schönkirchen, Germany; Consorci Parc de Salut MAR de Barcelona, SPAIN

## Abstract

**Introduction:**

For optimal treatment of femoral fractures, it is essential to understand the anatomical antecurvation of the human femur. Recent clinical studies have highlighted the problem of distal anterior encroachment or even perforation of the nail tip. The aim of this study was to accurately describe the femoral antecurvation in a large cohort. Another objective was to identify the most important influences on femoral antecurvation, such as age, femur length, gender and ethnicity.

**Methods:**

A three dimensional modelling and analytical technology was applied for the analysis of 1,232 femurs. Individual femoral antecurvation was precisely computed to determine whether gender, femur length, age, ethnicity or body mass index influence the radius of curvature (ROC).

**Results:**

The calculated mean ROC for all femurs was 943 mm. The lowest ROC of 826 mm was found in female Asian femurs. A regression analysis demonstrated that age and femur length could predict the variability of the curvature, with femoral length as most powerful predictor. A matched pair subgroup analysis between Asians and Caucasians could not show any significant differences of ROC values.

**Conclusions:**

The mean radius of the femoral antecurvation may be smaller than previously reported revealing a significant mismatch between the actual individual anatomy and existing implants. In opposite to existing literature, this study suggests, that antecurvation differences between various ethnicities may exclusively be attributed to differences in femoral length and age. The findings of this study may be found helpful in the development of novel designs for intra- and extramedullary implants.

## Introduction

For optimal treatment of femoral fractures, it is essential to understand the anatomical antecurvation of the human femur. This has been the subject of research for at least 80 years [1–6]. The first developments were in the 1940s, when straight steel nails were introduced by G. Küntscher [1]. Further developments led to highly refined intramedullary implants, such as the modern cephalomedullary nails [7,8]. However, there may still be room for improvement, especially in the anterior nail curvature for elderly patients. The radius of curvature (ROC) of most currently available nail designs ranges between 1500 and 2000 mm. Recent clinical studies have highlighted the problem of distal anterior encroachment or even perforation of the femur by the nail tip, especially in Asian patients—due to their shorter femurs with increased antecurvation [4,9–12].

Despite the extensive research, the results vary over a relatively large range, from 885 to 1446 mm [2,4,5,13,14]. Due to the small number of patients [3,5] and lack of data [4,13], no reliable conclusion may be drawn on differences in antecurvation between Asian and Western populations.

In this present study, three dimensional modelling and analytical technology (Stryker Orthopaedics Modelling and Analytics, SOMA) [15] was applied to a large CT dataset consisting of 1,232 femurs to accurately describe the femoral antecurvation. A refined measuring methodology was used to identify the most important influences on femoral antecurvation. The influence of age, femur length, gender and ethnicity was tested. Moreover, for the Asian population it is often hypothesised that there may be a significantly higher antecurvation when compared with Caucasian femurs.

## Methods

**Fig 1:**
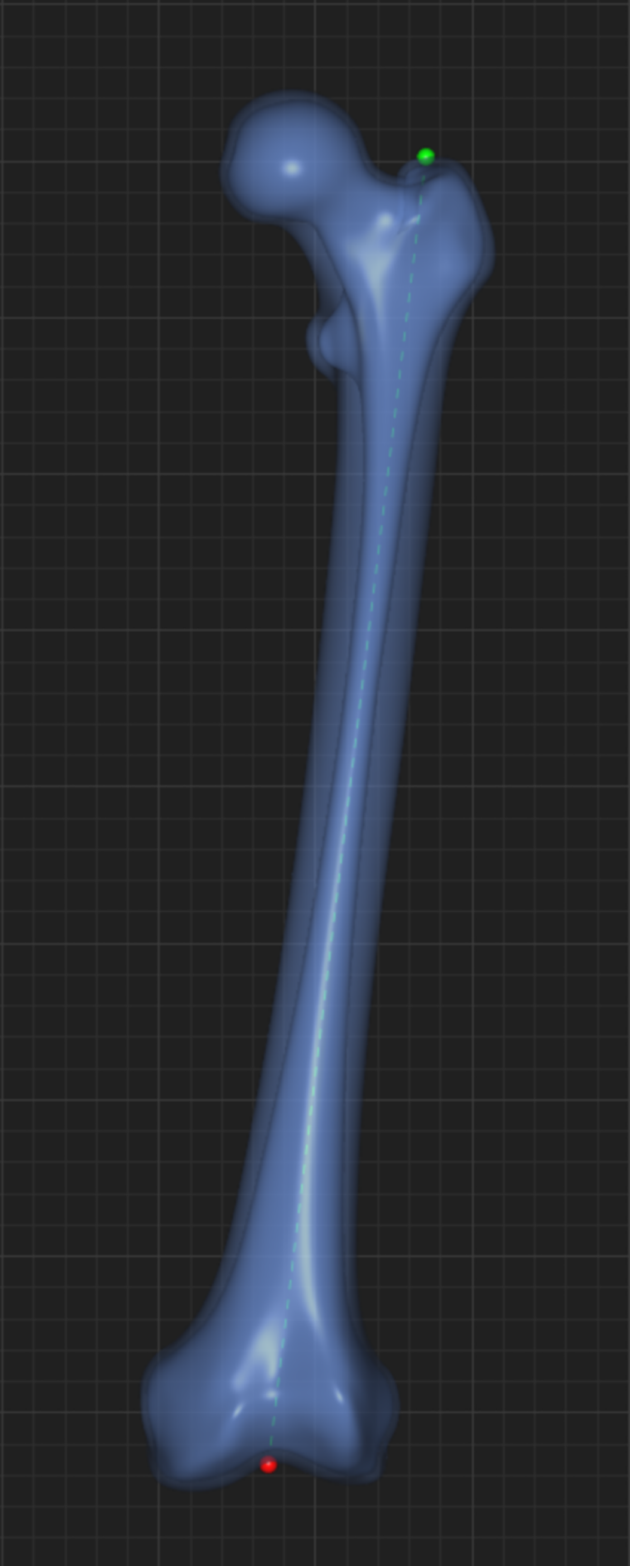
Shows the correlation between femur length and ROC for Asian and Caucasian femurs (n = 1,187).

SOMA was first developed in alliance between the Department of Orthopaedics and Sports Medicine of the Technical University of Munich, Germany, and Stryker Trauma GmbH (Kiel, Germany). [15] The CT datasets had all been obtained for medical reasons: in 20% in trauma patients, in 70% for CT angiography and in 10% for other indications (median pixel spacing: 0.78 mm, median slice spacing: 1.00 mm). The CT raw data were retrospectively acquired by Stryker Trauma GmbH between 2008 and 2017. Fractured, grossly deformed (tumorous, post osteomyelitis, post-traumatic) and implant-containing femurs were excluded in advance. Inclusion criteria was a fully scanned femur without the above-mentioned limitations, no radiological artefacts. In a first step all CT datasets were segmented using a standard software employing a standard protocol (MeVisLab and Materialise Mimics). [15] The segmentation results in precise 3D models representing both the bone surface and the cortico-cancellous interface. On the basis of these 3D models, SOMA offers the possibility to define landmarks, distances, angles, diameters and curves defined on a template femur. Measurements defined on this 3D femur template were transferred to each of the individual datasets (Fig 1). This procedure gives precise and reproducible results [15].

For the correlation analysis, 1,232 left femurs were used. For the matched pair comparison of Asian and Caucasian femurs, both left and right femurs were used to generate a larger sample size of 349 pairs. The results of earlier publications demonstrate significant individual symmetry in paired femurs [16]. Consequently, left and right femurs were taken in the Caucasian datasets as well as in the Asian datasets, rather than using two femurs from one individual.

### Determination of femoral length

The femoral length was defined as the distance measured from the tip of the greater trochanter to the saddle point of the intercondylar region at the distal femur in a straight line, referred to as the femur long axis, without correction of the existing individual curvature. The actual length along the anterior edge may already be understood as an expression of the individual curvature. It was therefore not separately measured (Fig 1).

### Determination of the radius of anterior-posterior curvature (ROC)

**Fig 2:**
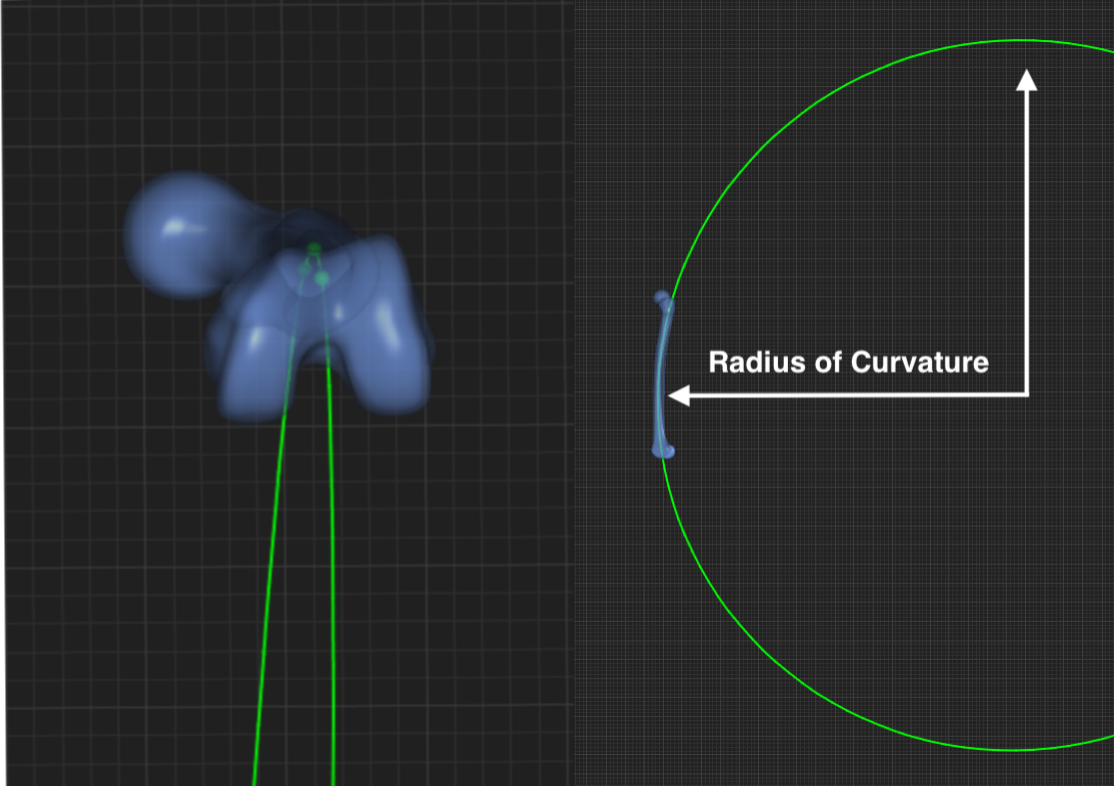
(left) shows an axial view of the femoral curvature as a section of the created 3D circle; the plane centres are represented by green dots–(right) shows the femur, portraying one section of the created 3D circle which was used to measure ROC.

To obtain the ROC, three planes where placed along the femur at 20%, 50% and 80% of the total femoral length perpendicular to the long axis, measured as described above. In each of these planes, the centre of the cancellous bone was defined by using the cortico-cancellous interface. These centre points of the three planes were connected, thus creating a section of a 3D circle with a specific radius. This represents the radius of femoral curvature; see Fig 2.

### Statistics

Continuous parameters are reported as means, medians and interquartile ranges (IQR). Categorical variables are reported as absolute counts with percentages in brackets. Those variables following a non-normal distribution, nonparametric approaches were employed for the statistical analyses. Spearman’s rho correlations were calculated for correlations between continuous variables. The Wilcoxon test was conducted to compare continuous variables between paired and independent groups. We used multiple linear regression analyses to determine the relative contribution of all metric variables such as age, femur length, sex, height, weight, isthmus diameter and proximal isthmus distance to the total variance of ROC. The significance level for all statistic tests was 95% (alpha = 0.05). Statistical analyses were all conducted using IBM SPSS V21 (IBM Corp., NY, USA).

This study has been approved by the local ethics committee.

## Results

### Statistics of meta data

The correlation analysis was based on 1,232 CT datasets of adult femurs. 820 (67%) of these femurs were of Caucasian origin, 369 Asian (30%), 28 African (2%), 14 Middle Eastern (1%) and 1 without known origin. The mean age was 64 years (n = 1,146, range 18–109 SD). Fifty-two percent (n = 636) of the subjects were male. The data on height, weight and thus the BMI were available in 926 of the subjects (75%). Mean BMI was 24.9 (range 13.3–54, SD). For a detailed overview of gender, age, BMI and height in relation to the ethnicities see Table 1.

**Table 1:** Gives an overview of the distribution of gender, age, BMI and height in relation to ethnicity.

Ethnicity	Gender (n = 1,232)	Age, mean+/-SD, (range), years (n = 1,146)	BMI mean+/-SD, (range), sqm/kg (n = 926)	Height mean+/-SD, (range), cm (n = 926)
**African**	male = 14	60.64 +/- 11.4, (41–78)	25.6 +/- 3.1 (20–33)	171 +/- 6.1 (160–181)
	female = 14	56.64 +/- 23.9, (20–85)	27.6 +/- 5.6 (19–39)	161.5 +/- 7.2 (147–172)
	n = 28	58.6 +/- 18.1	26.6 +/- 4.5	166.3 +/- 8.1
**Asian**	male = 177	59.1 +/- 17.9, (18–91)	24.1 +/- 3.3 (16–34)	169.8 +/- 7 (149–182)
	female = 192	69.2 +/- 18.7, (19–96)	22.2 +/- 4.1 (13–39)	152.6 +/- 7.5 (130–169)
	n = 369	64.5 +/- 18.9	22.9 +/- 3.9	159 +/- 11.1
**Caucasian**	male = 433	63.7 +/-14.3, (19–109)	26.2 +/- 4.9 (14–53)	175.1 +/- 7.6 (154–199)
	female = 385	65 +/- 15.5, (19–96)	26.5 +/- 6.2 (14–55)	164.8 +/- 7.6 (142–192)
	n = 818	64.3 +/- 14.9	26.3 +/- 5.6	170 +/- 9.2
**Middle Eastern**	male = 12	50 +/- 13.4, (31–77)	27.1 +/- 3.6 (23–33)	174.6 +/- 7.6 (160–186)
	female = 2	50 +/- 1.4, (49–51)	27 +/- 2.6 (25–29)	154 +/- 5.7 (150–158)
	n = 14	50 +/- 12.1	27.1 +/- 3.3	171.2 +/- 10.7

IQR, Interquartile Range, SD, Standard Deviation, mm, Millimetre, sqm, Square Metre, cm, Centimetre, kg, Kilogram

### Radius of curvature (ROC)

The calculated mean ROC was 943 +/- 212 mm for all left 1,232 femurs, with a median of 913 mm (IQR = 795–1059 mm). Female femurs exhibited significantly lower mean ROC values (895 mm) than male femurs (990 mm), p < 0.0001 (Wilcoxon test). The mean ROC value was significantly lower for the Asian subgroup (899 mm) than for the Caucasian subgroup (962 mm), p < 0.0001 (Wilcoxon test).

The female Asian cohort exhibited the lowest mean ROC—with 826 +/- 181 mm (n = 192). For male subjects, there was no significant difference in curvature for the different ethnicities; the mean value for male Asians was 987 mm, compared with 994 mm for male Caucasians (p = 0.42). There was however a significant difference between female Asians (826 mm) and female Caucasians (927 mm) (p <0.0001, see also Table 2).

**Table 2:** Gives an overview of radius of curvature (ROC) and femur length by gender and ethnicity.

	All Femurs (n = 1232)	Female (n = 594)	Male (n = 636)	Asian (n = 369)	Caucasian (n = 818)
**ROC, mean +/- SD, (range), mm**	943 +/- 212 (456–1852)	895 +/- 200 (457–1816)	990 +/- 213 (490–1853)	899 +/- 210 (457–1639)	963 +/- 209 (483–1853)
Median (IQR)	913 (795–1059)	864 (755–1009)	952 (840–1117)	872 (749–1021)	933 (816–1073)
**Femur Length, mean +/- SD, (range), mm**	420 +/- 31 (329–508)	402 +/- 26 (329–502)	437 +/- 25 (363–508)	398 +/- 27 (329–467)	430 +/- 28 (353–508)
Median (IQR)	421 (398–442)	401 (384–420)	437 (419–454)	397 (379–418)	430 (410–450)

IQR, Interquartile Range, SD, Standard Deviation, mm, Millimetre, ROC, Radius of Curvature

### Correlation analyses of ROC

**Fig 3:**
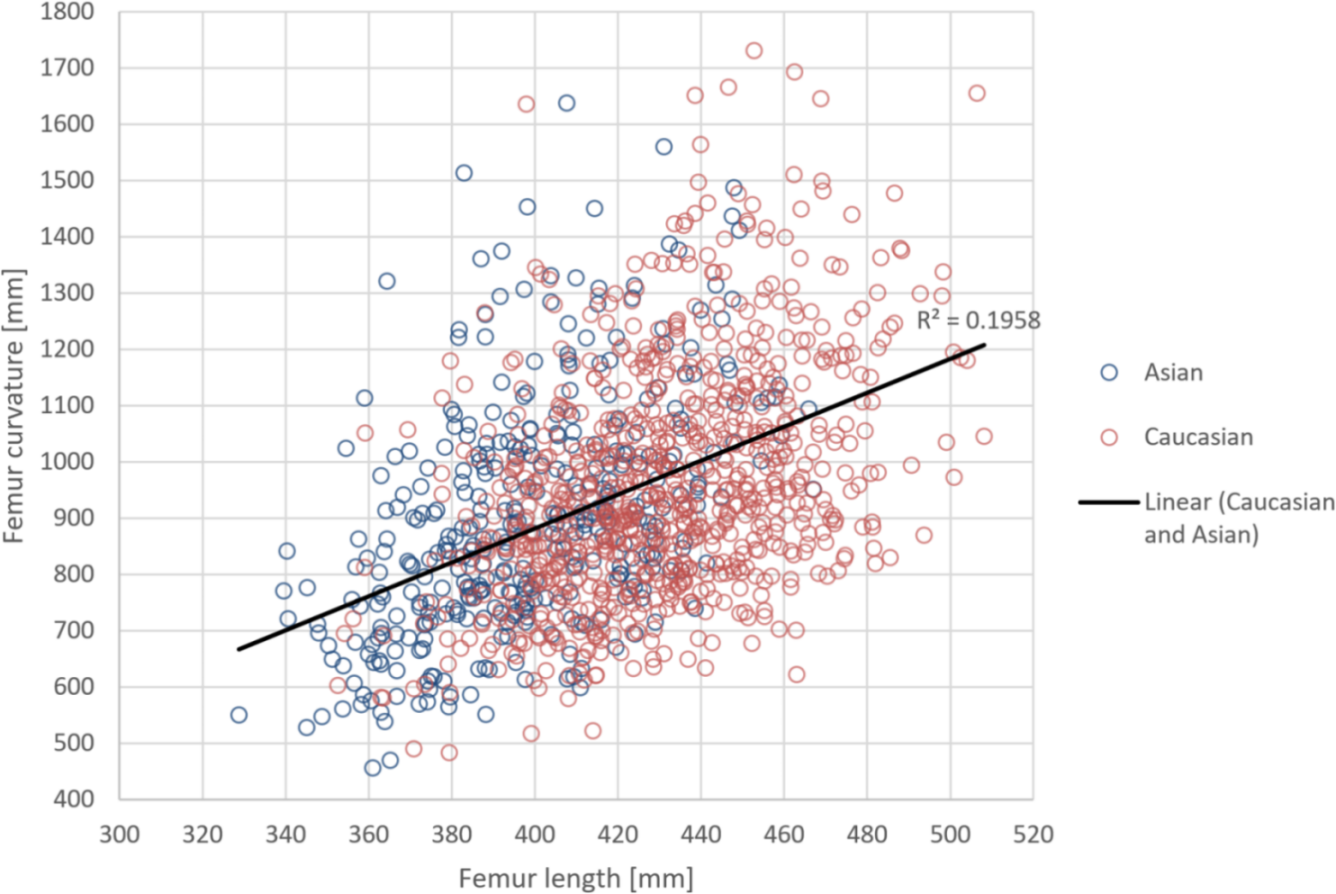
Depicts the definition of the femoral length, which was measured from the tip of the greater trochanter to the saddle point of the intercondylar region at the distal femur.

These analyses were performed exclusively with Caucasian and Asian femurs (n = 1,187). A moderate correlation was found between femur length and ROC (r = 0.44 with Spearman’s ρ, p < 0.0001, Fig 1). The linear regression line of ROC and femur length indicates mean ROCs ranging from 700 mm to 1200 mm, as shown in Fig 3.

Height and age were weakly correlated with curvature (r = 0.38 and r = 0.25 in Spearman’s ρ, p < 0.0001). All other meta data—such as weight, gender and ethnicity—exhibited weak to absent correlations to curvature.

A stepwise multiple regression analysis demonstrated that femur length, age, weight and isthmus diameter explain 26% of the variability of the curvature (p < 0.001, adjusted r^2^ = 0.26). All other variables had no significant impact on ROC. The unstandardized beta was for femur length 3.367 (CI 2.922–3.812), age -1.904 (CI -2.74 - -1.069), weight -1.403 (CI -2.157–0.650) and isthmus diameter -7.138 (CI -12.871 - -1.4), respectively. This means that with every millimetre of femur length, the ROC will grow by 3.4 mm, and with every additional year of age, the ROC will decline by 1.9 mm. The estimate of the isthmus diameter does not appear very accurate due to the large confidence interval.

Femur length was the strongest independent predictor in ROC—with adjusted r^2^ = 0.22 and p < 0.001 and a standardized Coefficients Beta of 0.496.

### Matched pair analysis

Asian and Caucasian left and right femurs were matched to achieve a homogenous subgroup with respect to femur length and age. Thus, each Caucasian femur was matched with a fitting Asian femur with a similar femur length +/- 28mm and age difference of +/- 2 years. The subgroup consisted of 698 femurs (Asian n = 349, Caucasian n = 349); the mean age of the Asians was 63.5 +/- 17 years, and the mean age of the Caucasians 63.5 +/- 17 years. The mean femur length of Asian femurs was 407 +/- 22 mm; the mean femur length of Caucasian femurs was 409 +/- 21 mm). The mean ROC for Asians was 911 +/- 204 mm, for Caucasians 888 +/- 182 mm (no significant difference, Wilcoxon test, p = 0.3).

## Discussion

The changes in the design of intramedullary nails after 1940 [1] indicate the importance of anterior bowing. Femoral antecurvation is an important factor in any precise analysis, as it may influence complications such as periprosthetic fractures, penetration and anterior encroachment [12,17,18]. In the present study, a refined measuring methodology was used with a large database containing 1,232 CT scans of femurs, in order to obtain individual data on femoral antecurvation (ROC = radius of curvature). Earlier studies showed that such automated morphometric analyses are highly accurate, with a general deviation of less than 2 mm compared to manually set landmarks [15]. The large database allowed the reliable investigation of ethnic and gender differences for the first time. The matched-pair comparison of Asian and Caucasian femurs showed no significant differences between Asian and Caucasian populations in terms of ROC. Correlations were found between age, femur length and ROC.

The results show a mean ROC of 943 mm within the entire population. The radius of curvature of the long femoral nails available today for intramedullary stabilization femoral fractures ranges from 1500mm to 3000mm [11,19] and does not fit our results to a great extent.

On the one hand, the results differ from a study by Schmutz et al. [3] who investigated mixed samples of Asian and Caucasian femurs (885 mm) using 3D bone models based on CT scans. On the other hand, the mean ROC of the Caucasian sample (963 mm) in the current study was close to the mean ROC of the Caucasian sample (974 mm) presented by Schmutz et al. [3]. The mean ROC in our population was much smaller than values collected in earlier surveys using different measuring methods (1090–1523 mm) [5,13,14,20,21]. Only Karakas et al. [22] reported even smaller results for mean ROC, based on data from 104 femurs of Caucasians living in Anatolia. A significant difference between the mean ROC of the female group (894 mm) and the male group (990 mm) was found in the current study. Some of the earlier studies gave different results [5,22], probably due to small sample sizes. However, a significantly smaller ROC in female femurs was reported by Maratt et al. [14] and by Egol et al. within their Caucasian subgroup [13].

Besides gender-specific differences, comparison of Asian and Caucasian femurs showed significantly smaller ROCs in the Asian population. Small ROCs in Asian populations have been described earlier [3,14]. In the current investigation, female Asian femurs had the smallest ROC of all groups and a significantly smaller ROC than female Caucasian femurs. Femoral curvature did not differ significantly between male Asians and male Caucasians.

Analysis of variables such as BMI, gender, ethnicity, femur length and age, showed that there was a moderate correlation between femur length and ROC, with increasing radius of curvature in longer femurs. Moreover, there was a weak correlation between age and ROC, corresponding to decreasing radius of curvature in older subjects. Maratt et al. [14] found similar correlations, although other studies did not [3,13,21]. Karakas et al. reported decreasing ROC in older femurs within their female population. [22]

Our regression analysis showed that femur length and age predict the variability of femoral curvature. These results should be compared with earlier findings by Schmutz et al. [3].

In a subgroup-analysis of this study, a group consisting of 349 Asian and Caucasian femur pairs was generated that was matched in age and femur length. In this comparison, no significant difference in curvature was found between Asian and Caucasian femurs. The current study suggests that the previously reported differences in ROC between different ethnicities [3] may be exclusively attributed to differences in femur length and age.

Schmutz et al. [23] reported “a weak to moderate correlation (r = 0.39) between height and ROC”–matching our findings (r = 0.37)—and suggested that further meta data such as weight could improve the prediction of ROC.

A correlation between femur length and radius of curvature (r = 0.38) has been shown before by Maratt et al. [14], with data from a large dataset of 1,961 patients. In the current study femur length and age (r = 0.44 and r = 0.25) displayed moderate and weak correlations to ROC, respectively. The femur length showed a more precise correlation than height alone (r = 0.37).

The regression analysis demonstrated that age and femur length explain 22% of variability in ROC as significant predictors.

In contrast to the correlations found by Maratt et al. [14], this study suggests that besides the femur length, the age of the patient is a significant factor which influences the ROC. As their values for radius of curvature were clearly different (mean 1120 mm), it seems questionable whether their methodology was as accurate as that in the current study. Their measurements were performed on the basis of 2D CT Scans with a low resolution, in contrast to our 3D measurements with a pixel spacing of 0.78 mm and slice spacing of 1.00 mm. For femur length, they found similar results to ours, which implies that the three dimensional structure of the medullary canal is complex. This may indicate that 3D measurements are more suitable.

The results of the present study are quite different from those found in earlier investigations of femoral anatomy. With one exception [3], all former studies used different and less accurate methods to measure the radius of curvature. Therefore, direct comparisons may not be applicable. CT slices, plain radiographs and multi-planar reconstructed CT views are simplified reproductions of the diverse 3D structure of the human femur. Measuring multiple aspects and characteristics in already simplified representations may be a flawed approach. A 3D model generated by modern software based on CT datasets, as performed in the present study, has been demonstrated to be more accurate and reproducible [15].

Besides substantial differences in the methods, the patient cohorts of the compared studies are very different. With one exception (Maratt et al. [15]; n = 1,961), all other studies had much smaller sample sizes, ranging from 14 to 200 subjects [5,13,20,21,23–29]. Some publications just looked at a single ethnic group [5,26]; some compared up to four groups, but with very small sample sizes [3], while other studies totally failed to report ethnicity [24,25], for an overview, see Table 3. The advantages of the present study lie in the greater accuracy and the comparably high sample size.

**Table 3:** Shows the comparison of radius of curvature [mm] measured in this and other studies.

Author	Harper[20]	Maratt[14]	Egol[13]	Buford[5]	Karakas[22]	Schmutz[3]	Thiesen
**Measurement Method**	X-Ray	CT Scan 2D	Digital Photography	CT 3D Cadaver	CT Scan 2D	CT 3D Automated	CT 3D Automated
**Sample Size**	14	1961	474	24	104	90	1232
**Overall ROC**	1114	1120±260	1200 ± 360	1446 ± 397	-	885	943±212
**Female**	-	1085 ± 245	1160 ±340	-	779 ± 267	-	895±200
**Male**	-	1170± 273	1220 ±360	-	759±269	-	990±213
**Asian**	-	1011 ± 230	-	-	-	787	899±210
**Caucasian**	-	1104 ± 243	-	-	-	974 ± 167	963± 209
**African**	-	1222 ± 327	-	-	-	-	-

### Limitations

The comparison of ROC between the Asian and Caucasian cohort was restricted by the slightly different age distribution in these groups. Therefore, an additional matched pair analysis was carried out, revealing no significant differences between the two subgroups In this study we assumed a general symmetricity of left and right femurs. Scientifically, this has yet been shown to be true at the proximal femur. [16] Although it seems legitimate to generalize this on the entire femur, our assumption may still be a possible source of error. The small numbers of African as well as Middle Eastern subjects may be limiting the generalization of the findings on all existing ethnical groups. A total of 926 femurs was considered for linear regression analysis regarding height and weight. The data on height and weight were unavailable for 306 patients. Data on age were unavailable in 86 cases.

## Conclusions

The mean radius of the femoral antecurvation may be smaller than previously reported according to the findings of this study. A matched pair analysis comparing Asian and Caucasian femurs revealed no significant differences with regard to femoral antecurvation. Femoral length turned out to be the most powerful predictor of femoral antecurvation. The findings of this study may be found useful in the development of novel designs for intra- and extramedullary implants.

## Supporting information

S1 File S1 File contains the underlying raw data used in this study. The data file contains a legend explaining the meaning of each data column and the measurements made by SOMA and the authors.
(XLSX)
